# Do Patients Receiving GLP-1 Receptor Agonists for Weight Loss Require Structured Dietetic Care? Lessons from Metabolic and Bariatric Surgery (MBS) Pathways

**DOI:** 10.3390/nu18162643

**Published:** 2026-08-13

**Authors:** Vignesh Balasubaramaniam, Megan Scobie, Mary O’Kane, Yitka Graham, Andrew G. Robertson, Kamal Mahawar

**Affiliations:** 1 Wrexham Maelor Hospital, Wrexham LL13 7TD, UK; 2 Dietetic Department, Leeds Teaching Hospitals NHS Trust, Leeds LS1 9LF, UK; marywasokane@gmail.com; 3 Department of Surgery, South Tyneside and Sunderland NHS Foundation Trust, Sunderland SR4 7TP, UK; yitka.graham@sunderland.ac.uk (Y.G.); kmahawar@gmail.com (K.M.); 4 Helen McArdle Nursing and Care Research Institute, University of Sunderland, Sunderland SR6 0DD, UK; 5 Faculty of Biomedical Sciences, Austral University, Buenos Aires B1406, Argentina; 6 Faculty of Psychology, University of Anahuac, Mexico City 52786, Mexico; 7 Clinical Department of Surgery, Royal Infirmary Edinburgh, University of Edinburgh, Edinburgh EH16 4SA, UK; andrew.robertson6@nhs.scot

**Keywords:** GLP-1 receptor agonist, semaglutide, tirzepatide, obesity, dietitian, nutritional care, MBS, sarcopenia, micronutrient deficiency, weight regain

## Abstract

Background: Glucagon-like peptide-1 receptor agonists (GLP-1 RAs) and dual incretin therapies have significantly advanced obesity management by facilitating clinically significant weight loss and enhancing cardiometabolic outcomes. Nevertheless, their growing application calls for careful consideration of nutritional and behavioural factors, including decreased dietary intake, gastrointestinal symptoms, loss of lean mass, micronutrient deficiencies, and potential weight regain following treatment cessation. Methods: This narrative review synthesises evidence from clinical trials, observational studies, reviews, professional guidelines, and MBS nutrition protocols to evaluate the role of structured dietetic care in adults receiving GLP-1 RAs or related incretin-based therapies for weight management. MBS pathways provide a comparative framework for investigating how established principles of nutritional assessment, monitoring, and dietetic follow-up may inform pharmacological treatments of obesity. Results: The literature indicates that pharmacological weight-loss treatments should not be administered solely as prescription-based treatments. Structured dietetic care can facilitate nutritional assessment, personalised dietary interventions, the management of gastrointestinal symptoms, protein optimisation, behavioural counselling, physical activity support, and long-term weight maintenance. MBS pathways illustrate the role of dietitians in pre-, peri-, and post-major weight-loss interventions through assessment, guidance on supplementation, dietary progression, biochemical monitoring, and long-term maintenance. Although these principles require adaptation rather than direct application to GLP-1 RA pathways, they offer a valuable system for integrating dietitians into comprehensive obesity pharmacotherapy. Conclusions: Structured dietetic care should be regarded as a fundamental component of the pharmacological management of obesity, especially as GLP-1 RAs and incretin-based therapies become more prevalent. Dietitians are well positioned to help optimise nutritional adequacy, preserve lean mass, manage gastrointestinal symptoms, and support sustained weight maintenance. Future research should investigate effective models for delivering dietetic care alongside incretin-based therapies, including strategies to identify patients who need more intensive support.

## 1. Introduction

Obesity is a chronic, multifactorial disease characterised by excessive fat accumulation that adversely affects organ function and metabolic health [1]. Historically, MBS has been the most effective intervention for attaining significant and sustained weight loss in patients with severe and complex obesity [2]. Recently, glucagon-like peptide-1 receptor agonists (GLP-1 RAs) and dual incretin-based therapies have markedly transformed the therapeutic landscape. Clinical trials involving Semaglutide and Tirzepatide have demonstrated clinically meaningful weight reduction over extended follow-up periods, narrowing the gap between pharmacological and surgical obesity treatments [3,4,5]. Current clinical guidelines recognise the importance of pharmacotherapy as part of a comprehensive approach to obesity management, generally recommending its use alongside reduced energy intake, physical activity, and behavioural support, rather than as an isolated intervention [1,6,7,8].

The effectiveness of these agents is closely linked to their impact on appetite regulation, satiety, eating behaviour, and overall energy intake. Semaglutide has been shown to reduce appetite, improve eating control, and lower ad libitum energy intake, while Tirzepatide results in substantial weight loss through combined incretin receptor activity [5,9]. Although these mechanisms are therapeutically beneficial, they also present nutritional challenges. These include a significant reduction in intake that may decrease not only energy intake but also protein, fibre, fluid, vitamin, and mineral intake [9,10,11,12].

The rapid adoption of GLP-1 RA therapy has raised important questions regarding service design and long-term clinical support. Data indicate increasing use among individuals without diabetes, while real-world discontinuation and re-initiation patterns suggest variable persistence with therapy [13,14]. Evidence from withdrawal studies and systematic reviews further demonstrates that the cessation of pharmacological weight management treatment is frequently followed by weight regain and attenuation of cardiometabolic benefits [15,16,17,18,19]. These findings suggest that a care model focused primarily on prescription and dose escalation may be inadequate for the long-term management of obesity.

Despite the expanding use of GLP-1 RA and related incretin-based therapies, structured nutritional and behavioural support is not consistently integrated within pharmacological obesity pathways. Consequently, many patients may initiate, continue, or discontinue treatment without adequate dietary advice regarding dietary quality, protein intake, gastrointestinal symptom management, micronutrient adequacy, physical activity, and long-term weight maintenance. This gap is crucial given the increasing access through private providers and direct-to-consumer services, where treatment may occur outside traditional specialist obesity pathways and without consistent multidisciplinary monitoring. Insufficient nutritional counselling may limit patients’ understanding of treatment expectations and side effects, reduce opportunities for timely follow-up, and undermine the durability of weight-loss outcomes.

In MBS, nutritional care is an essential aspect of care and embedded into routine patient care. Established pathways include preoperative nutritional assessment, perioperative care, postoperative supplementation, biochemical monitoring, protein optimisation, and long-term follow-up [20,21,22,23,24,25,26]. Although the mechanisms of nutritional risk differ between surgery and pharmacotherapy, both approaches may be associated with reduced dietary intake, loss of lean body mass, gastrointestinal symptoms, behavioural vulnerability, and the need for long-term weight maintenance support [10,20,21,22,23,24,25,26,27]. This raises an important clinical question: should principles from MBS nutritional care, which is often dietitian-led, be adapted to GLP-1 RA treatment pathways?

This narrative review seeks to assess the impact of structured dietetic care on patients receiving GLP-1 RA or related incretin-based therapies for weight loss. Additionally, it explores how established principles from MBS dietetic pathways can inform nutritional assessment, intervention, and follow-up in the context of pharmacological obesity treatment.

## 2. Methodology

This review utilised a structured narrative approach to synthesise current evidence on nutritional and dietetic care in adults undergoing GLP-1 RA therapy or related incretin-based pharmacotherapy for obesity. In healthcare, a narrative review is a rigorous and structured approach that allows exploration of an intervention, offering a broad insight and interpretation across identified literature, comparing findings across studies, and identifying what research gaps exist along with situational contexts that can provide a greater understanding, inform practice and guide future research [28].

MBS pathways serve as a comparative framework to explore how established principles of nutritional assessment, monitoring, and dietetic follow-up may inform the pharmacological treatment of obesity. The method comprised three key stages: literature identification, relevance screening, and thematic synthesis.

**Figure 1:**
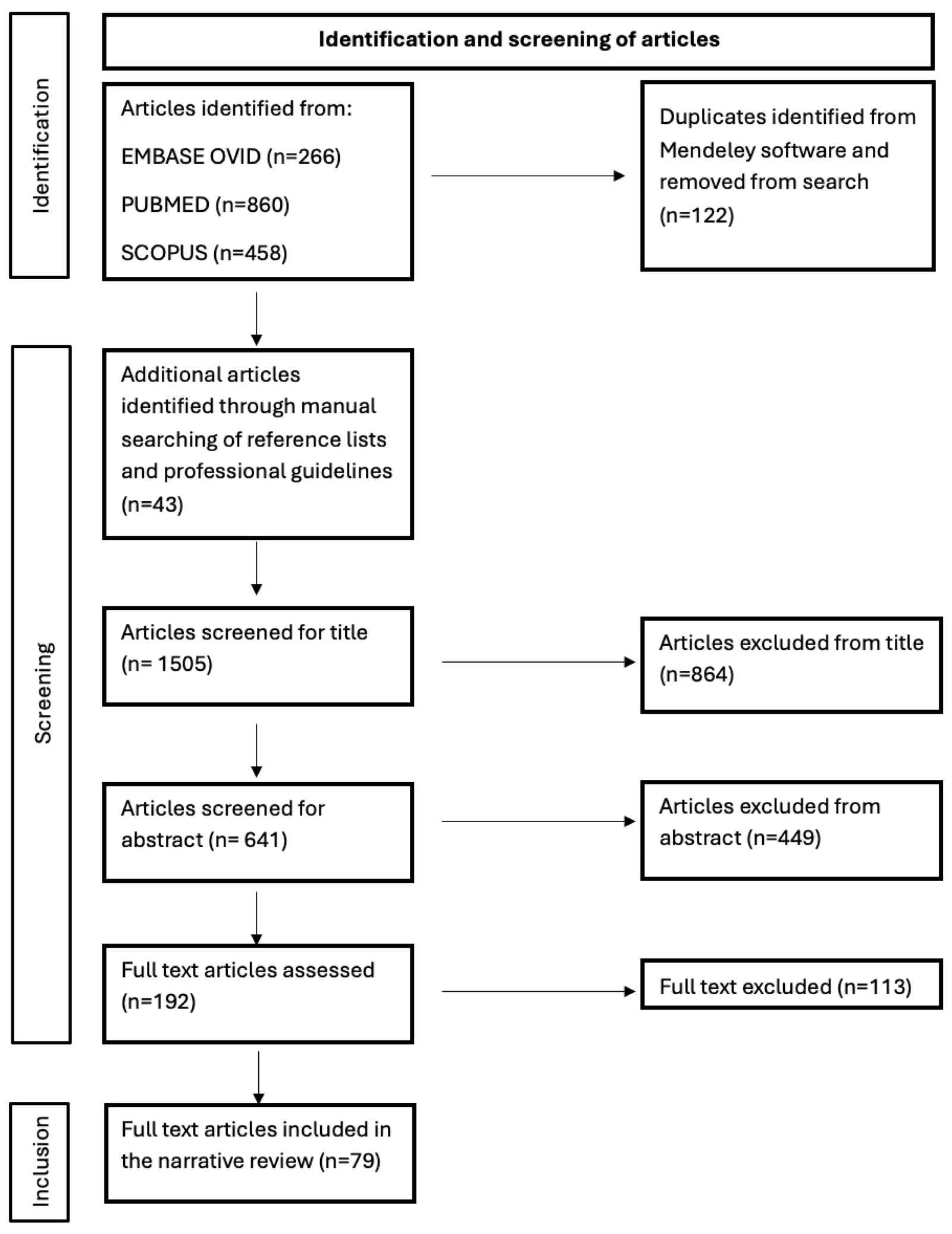
A flow diagram of the identification, screening and included articles.

A comprehensive literature search was conducted in May 2026 across PubMed, Embase and Scopus. This was then supplemented by direct searches on Google Scholar and the by-hand searching of reference lists. The search strategy integrated Medical Subject Headings (MeSH), where applicable, with free-text keywords related to incretin-based therapies, obesity, nutritional outcomes, and dietetic care. The drug-related search terms used were “glucagon-like peptide-1 receptor agonists,” “[MeSH],” “GLP-1 receptor agonist,” “semaglutide,” “liraglutide,” “tirzepatide,” “antiobesity medication,” and “GIP/GLP-1 dual agonist.” Nutritional and dietetic search terms used were “dietitian,” “[MeSH],” “micronutrients,” “macronutrients,” “nutrient deficiency,” “medical nutrition therapy,” “nutrition assessment,” “dietetic care,” “nutritional counselling,” “nutrition support,” “monitoring,” and “follow-up.” The population and comparative pathway terms used were “obesity,” “[MeSH],” “weight loss,” “MBS,” “bariatric”, and “metabolic surgery.” These terms were combined using Boolean operators (“AND” and “OR”) to ensure a comprehensive yet focused search strategy. The search was then limited to human-only articles, full text articles and those in English before further screening. Exclusion criteria included animal studies, conference abstracts, articles not published in English for which a translation was unavailable, and articles not relevant to the review question. Duplicates were identified using Mendeley Reference Manager and were removed before further screening. A flow diagram is presented in Figure 1, which demonstrates the different stages of our screening process and the number of articles removed at each stage.

The search primarily covered publications from January 2016 to April 2026 to capture contemporary evidence on the current types of GLP-1 RAs. However, MBS nutrition guidelines and professional society recommendations published before this period were retained if they remained clinically relevant to current practice. Additional records were identified through the manual screening of reference lists from key reviews, clinical trials, and professional guidelines, including guidance from the National Institute for Health and Care Excellence (NICE), British Obesity & Metabolic Specialist Society (BOMSS), American Society for Metabolic and Bariatric Surgery (ASMBS), European Society for Clinical Nutrition and Metabolism (ESPEN), and other MBS and obesity societies.

Clinical trials evaluating the dietitian’s role in GLP-1 RA therapy do not yet exist. However, we considered evidence as direct if it was derived from GLP-1 RA trials or from observational cohort studies of treated populations. Evidence was considered indirect when it was extrapolated from literature on broader topics such as obesity, MBS, discontinuation, nutritional outcomes, gastrointestinal symptoms, nutritional risk, and eating disorders. We also used practice-based guidelines and expert opinion from MBS to inform clinical considerations.

Articles were selected based on their relevance to three primary areas of interest: (1) the nutritional implications and support needs associated with GLP-1 RA therapy, encompassing dietary intake, gastrointestinal symptoms, weight regain, micronutrient and macronutrient status, (2) current recommendations and evidence for dietetic care during GLP-1 therapy; and (3) established practices for nutritional assessment, monitoring, and dietetic follow-up within MBS pathways. Supplementary Table S1 provides a concise summary of the key literature we included to identify common themes, areas of agreement, evidence gaps, and implications for clinical practice. Full-text articles were excluded if the focus of the article was not on weight loss but rather diabetic control or diabetic complications, or if it was felt the data from the article were unclear. Special emphasis was placed on evidence detailing the clinical role of dietitians. Given the narrative nature of this review and the heterogeneity of the available evidence, formal systematic review methods, such as PRISMA reporting and quantitative meta-analysis, were not employed. Nonetheless, a structured approach to literature selection and thematic synthesis was adopted to minimise selection bias and maintain the review’s focus on the role of dietetic care in the pharmacological management of obesity.

## 3. Results

### 3.1. Nutritional Consequences of GLP-1 Receptor Agonist Therapy

The primary nutritional consequence of GLP-1 RA therapy is a reduction in dietary intake, which is intentional, as appetite suppression and enhanced satiety are key mechanisms for weight loss. Direct evidence from a study on once-weekly Semaglutide demonstrated that treatment led to reduced appetite, improved eating control, and decreased energy intake, indicating a direct impact on eating behaviour [9]. However, as pharmacological weight loss becomes more effective, distinguishing between therapeutic caloric reduction and nutritional inadequacy is becoming increasingly crucial. Reviews consistently stress the importance of maintaining dietary quality, protein intake, hydration, and micronutrient adequacy during therapy [10,29,30,31].

Macronutrient adequacy constitutes a significant concern. Several reviews indicate that reduced appetite, early satiety, and gastrointestinal symptoms may hinder patients from achieving adequate protein intake, particularly during dose escalation or periods of nausea [12,32,33,34]. This issue is clinically significant because weight loss is typically accompanied by some loss of fat-free mass. Although the loss of lean mass may partly reflect an adaptation to reduced body weight, excessive loss may be detrimental to patients with sarcopenia, frailty, older age, or low baseline muscle reserves [30,35,36,37,38].

The literature on muscle health advocates for a balanced perspective. Linge et al. advise that GLP-1 RA-associated reductions in muscle mass should not be automatically deemed maladaptive, but suggest that body composition, muscle function, and individual patient risk should be considered rather than body weight alone [39]. The nutrition and sarcopenia literature highlights that protein intake and physical activity, particularly resistance exercise, are important anabolic stimuli for preserving muscle during weight loss [40,41,42]. However, the high-protein literature also indicates that protein recommendations should be individualised rather than applied simplistically, as very high protein intake during weight loss may have complex metabolic consequences in certain populations [43].

Micronutrient deficiency is less well documented in GLP-1 RA users than in patients post-MBS, but emerging reviews suggest that the risk is biologically plausible and clinically relevant, as reduced intake may compromise the consumption of micronutrient-rich foods [10,37]. Unlike MBS procedures, GLP-1 RA therapy does not alter gastrointestinal anatomy or affect micronutrient absorption. Therefore, the nutritional risk arises from reduced intake, dietary restriction, food aversion, gastrointestinal adverse effects, or inadequate diet planning [10,37], and not reduced absorption, as is the case with some MBS procedures.

Gastrointestinal adverse effects are among the most common barriers to treatment tolerance. Systematic reviews and clinical guidance identify nausea, vomiting, diarrhoea, and constipation as frequent adverse effects of GLP-1 RAs and dual incretin therapies [32,34,44,45,46,47]. In the STEP 1 trial by Wilding JPH et al., gastrointestinal events were reported in 74.2% of participants receiving Semaglutide, compared with 47.9% in the placebo group [48]. Adverse events were classified as mild, moderate or severe according to their effect on daily activities. Transient, mild-to-moderate gastrointestinal disorders were the most frequently reported adverse events. More participants in the Semaglutide group than in the placebo group discontinued the assigned regimen after such events. In the STEP-UP trial, gastrointestinal adverse events were higher in the 7.2 mg group (70.8%) compared to the 2.4 mg group (61.2%) and placebo (42.8%) [49].

A systematic analysis by Bettge et al. compared gastrointestinal adverse events across short-acting GLP-1 RAs (Lixisenatide and Exenatide) and long-acting GLP-1 RAs (Dulaglutide, Albiglutide and Liraglutide) [44]. The authors found that nausea showed evidence of dose-dependency, particularly at higher doses across all types of GLP-1 RAs, with vomiting showing a similar trend, but the difference was not statistically significant. Long-acting agents are also associated with less nausea and vomiting but with more diarrhoea. Regression analyses have also demonstrated significant associations between nausea, vomiting and diarrhoea and withdrawal from clinical trials, both for any reason and due to adverse events, with stronger associations observed for adverse-event-related withdrawals.

These symptoms may lead to reduced nutritional intake, impaired hydration, and decreased treatment adherence, highlighting the potential need for dietary counselling and dietetic support to maintain nutritional adequacy during treatment.

Weight regain following the cessation of GLP-1 RA or GIP/GLP-1 RA therapy constitutes a significant factor in long-term obesity management. A study by Rodriguez PJ et al. reported that 53.6% of patients discontinued GLP-1 RA by 1 year, and 72.2% by 2 years [14]. Evidence from studies on treatment cessation indicates weight regain after withdrawal and potentially diminished metabolic benefits [15,16,17,18,19]. In the STEP 1 extension, participants treated with Semaglutide experienced a 17.3% mean weight loss at week 68, but regained 11.6 percentage points by week 120 after treatment withdrawal, with cardiometabolic improvements also reverting towards baseline for several variables [18]. Similarly, in SURMOUNT-4, participants achieved a 20.9% mean weight reduction after 36 weeks of Tirzepatide. Those switched to placebo subsequently regained 14.0% of their body weight, whereas those continuing Tirzepatide achieved an additional 5.5% reduction [15]. These findings suggest that pharmacological weight loss should not be considered a short-term intervention, and underscore the importance of structured dietetic and behavioural support to prepare patients for long-term weight maintenance during dose reduction or treatment discontinuation. Aside from the barriers in terms of cost and long-term safety of these drugs, there may also be reduced long-term efficacy due to receptor adaptation and tachyphylaxis.

### 3.2. Patients at Increased Nutritional Risk During GLP-1 Therapy

Patients at increased nutritional risk encompass those with sarcopenia or frailty, older adults, individuals with a history of MBS, those experiencing significant gastrointestinal symptoms, those with poor baseline diet quality, pre-existing nutritional deficiencies, a history of eating disorders, and those undergoing rapid or excessive weight loss [27,29,30,50,51,52].

Older adults warrant particular attention when undergoing GLP-1 RA therapy, as intentional weight loss may exacerbate loss of muscle mass and function if protein intake, physical activity, and resistance exercise are not effectively incorporated. Consensus guidelines define sarcopenic obesity by the concurrent presence of high body fat percentage and sarcopenia, characterised by reduced skeletal muscle mass together with impaired muscle strength or physical function, a condition particularly pertinent to older, frail, and chronically ill populations [53].

Reviews of sarcopenic obesity indicate that this combination of increased fat mass and reduced muscle function is linked to adverse clinical outcomes and necessitates integrated nutritional and physical activity strategies [41,54]. Although GLP-1 RA therapy is not inherently sarcopenic in otherwise healthy individuals, patients who are older, prefrail, sedentary or have poor baseline dietary quality may be more vulnerable to clinically significant muscle loss during weight reduction [39]. Smith et al. studied weight loss-induced decline in mean muscle mass by comparing a high-protein diet (defined as 1.2 g/kg/day) to a low-protein diet (defined as 0.8 g/kg/day) in women who were postmenopausal and living with obesity. They found that in this population, a high protein diet stopped decline in lean muscle mass by approximately 45% [43]. This highlights the need for early dietetic evaluation to identify nutritional risks, such as older age, to optimise protein intake, and encourage resistance-based physical training.

Patients with a history of MBS constitute another high-risk group. They may already require lifelong micronutrient supplementation and biochemical monitoring, with some procedures posing ongoing risks of deficiencies in iron, vitamin B12, folate, vitamin D, calcium, and trace elements [20,21,22,23,24,25,26]. In these patients, GLP-1RA therapy may be used to manage insufficient weight loss or weight regain after surgery; however, further pharmacotherapy-induced appetite suppression and reduced intake may exacerbate pre-existing nutritional issues [27,30]. Patients and their treating clinicians need to be aware of it. Once again, dietitians can help provide this education and mitigate the adverse consequences.

A comprehensive assessment is required for individuals with a history of eating disorders. Reviews of GLP-1 RAs in populations with eating disorders indicate potential benefits for binge-eating disorders. However, they also emphasise the limited evidence available for anorexia nervosa, bulimia nervosa, and avoidant/restrictive food intake disorder [51,55,56]. Therefore, the evidence should be interpreted with caution. For selected patients with binge-eating symptoms, appetite and reward-related mechanisms may be therapeutically beneficial, but patients with restrictive eating pathology, purging behaviours, or significant body image issues may require multidisciplinary assessment and monitoring before and during treatment [51,55,56].

A joint advisory and a review recommended screening patients for current or previous eating disorders, including binge-eating disorder, anorexia nervosa, bulimia nervosa and night-eating syndrome [31,54]. The effects of GLP-1-based therapies in these conditions remain uncertain and may vary between individuals, with the potential either to improve or worsen disordered eating behaviours [31,54]. Patients with a positive screening result or a history of an eating disorder should therefore be assessed by clinicians with expertise in obesity medicine and eating disorders before treatment is initiated. GLP-1-based therapy should generally be avoided in patients with an active restrictive eating disorder [31,54]. A simple screening tool such as the validated SCOFF questionnaire could be used to identify people who may potentially have an eating disorder and require specialist assessment about suitability for treatment with GLP-1 RA [57]. Most dietitians have a good understanding of eating disorders and can help identify patients who should not be given these medications, or those who need closer monitoring.

### 3.3. Nutritional Assessment and Dietetic Follow-Up in Current MBS Pathways

MBS pathways represent the most established model of structured dietetic care in the treatment of obesity. Within these pathways, dietitians are integral members of the multidisciplinary team, playing a key role throughout the preoperative, perioperative, and long-term postoperative phases of care. Current guidelines from many national and international bodies underscore the significance of nutritional assessment, the correction of pre-existing deficiencies, dietary education, postoperative supplementation, and long-term biochemical monitoring [20,21,22,23,24,25,26]. This emphasis reflects the understanding that substantial weight loss, dietary restriction, altered gastrointestinal anatomy, and food intolerance can contribute to nutritional vulnerability following surgery.

Prior to MBS, dietetic assessment is used to evaluate baseline dietary intake, eating patterns, nutritional knowledge, micronutrient status, protein intake, hydration, supplementation requirements, and readiness for postoperative dietary changes [20,21,22,23,25,26]. Dietitians also provide education on the expected staged dietary progression post-surgery, portion size, texture modification, protein prioritisation, fluid intake, avoidance of grazing behaviours, and the necessity for lifelong adherence to vitamin and mineral supplementation. This preoperative role is important because nutritional risk is often present before surgery, with many patients with obesity having pre-existing micronutrient deficiencies or poor-quality dietary intake before any weight-loss intervention [21,22,23,58].

In the early postoperative phase, dietitians guide patients through a staged dietary progression while monitoring tolerance, gastrointestinal symptoms, hydration, and adequacy of protein intake [21,22,24,25,26]. They also support adherence to micronutrient supplementation and help patients manage common issues such as nausea, vomiting, food aversion, dumping symptoms, constipation, and challenges meeting fluid or protein targets. In this context, dietetic care is both preventive and supportive, aiming to mitigate the risk of nutritional complications while identifying early symptoms that may necessitate biochemical testing, supplementation adjustment, or escalation to the broader multidisciplinary team.

Long-term dietetic follow-up is integral to MBS care, as nutritional risks extend beyond the immediate postoperative period. Guidelines recommend continued follow-up after MBS, including monitoring of micronutrient status, anaemia-related markers, bone health, supplement adherence and overall dietary adequacy. Closer review is particularly important for patients who become pregnant, regain weight, develop gastrointestinal symptoms, or struggle to attend follow-up or adhere to supplementation [21,22,23,24,25,26]. Dietitians thus contribute not only to nutritional safety but also to weight maintenance, behavioural relapse prevention, and the identification of late nutritional complications.

The MBS model illustrates that structured nutritional care is most effective when dietitians are integrated into the treatment pathway from the outset, rather than consulted only after complications arise. Although GLP-1 RA does not involve surgically altered anatomy or the same degree of malabsorption, several principles from MBS dietetic practice are potentially applicable. These include baseline nutritional assessment, risk stratification, protein optimisation, micronutrient monitoring, management of reduced intake and gastrointestinal symptoms, behavioural support, and planned long-term follow-up [10,20,21,22,23,24,25,26]. Therefore, the relevance of the MBS model lies not in equating pharmacotherapy with surgery, but in recognising that substantial weight loss interventions necessitate structured nutritional oversight, particularly in patients at increased risk of nutritional inadequacy.

### 3.4. Current Evidence and Recommendations for Dietetic Care During GLP-1 Therapy

Direct evidence assessing dietitian-led care specifically in the context of GLP-1 RA treatment remains scarce. A systematic review of GLP-1 RA trials involving adults with obesity but without diabetes found that dietitian involvement was inconsistent and often inadequately described, despite lifestyle interventions being a fundamental component of many studies [59]. For instance, the STEP 3 trial demonstrated that Semaglutide can be delivered alongside intensive behavioural therapy, but the structured support provided within a clinical trial may not reflect a clearly defined dietetic pathway in routine care [60]. Likewise, the STEP 1, STEP 5 and SURMOUNT trials incorporated organised lifestyle interventions alongside pharmacotherapy, but none were designed to isolate or independently assess the contribution of dietetic care [3,15,48].

NICE guidelines emphasise that pharmacological treatment for overweight and obesity should not be administered in isolation. The guidelines recommend that weight-management medications should be considered only after dietary, physical activity, and behavioural approaches have been initiated and evaluated, and that all medications should be used in conjunction with a reduced-calorie diet and increased physical activity [6]. Furthermore, NICE advises that when medications are prescribed, patients should receive information, support, and counselling on additional dietary, physical activity, and behavioural strategies [6]. Similarly, NICE has highlighted that Tirzepatide should be considered within weight-management services that include diet and exercise support. However, NICE also acknowledged that long-term diet and exercise support is not consistently available in primary care, underscoring the need to consider how structured dietetic input can be integrated into pharmacological obesity treatment pathways [8].

The Academy of Nutrition and Dietetics advocates integrating dietetic care into obesity pharmacotherapy pathways. The Academy emphasises that dietitians are instrumental in providing medical nutrition therapy for individuals using incretin-based therapies as part of comprehensive obesity care [61,62]. This includes nutritional screening and assessment, support for medication-related dietary issues, guidance on nutrient-dense eating patterns, promotion of physical activity, support for positive body image, and strategies to help preserve lean body mass during weight loss [61]. These recommendations align with the broader literature, which suggests that pharmacological weight-loss treatment should be accompanied by structured nutritional and behavioural support rather than delivered as medication alone [36].

Recent advisory and literature reviews increasingly advocate integrating nutritional support into GLP-1 RA treatment. Joint advisory documents emphasise the importance of dietary quality, adequate protein, fibre, hydration, micronutrients, and physical activity as priorities during therapy [31,52]. Reviews of anti-obesity medications and incretin-based therapies similarly recommend baseline nutrition assessment, the ongoing monitoring of intake and symptoms, attention to body composition, and referral to dietitians for patients at high risk or with inadequate intake [29,37,38,63].

Professional statements and the dietetic literature position dietitians as crucial members of the obesity care team in the era of pharmacotherapy. Dietitians can assess dietary intake and diet quality, identify risk for disordered eating, support nutritional targets, manage gastrointestinal symptoms, counsel on physical activity and resistance exercise in collaboration with the wider team, and support long-term maintenance when medication is stopped or paused [61,64,65,66,67]. Qualitative research involving registered dietitians highlights the critical need for explicit communication regarding nutrition, lifestyle, and medication expectations. This suggests that dietetic input could enhance patient comprehension and treatment experiences [68]. Nevertheless, the evidence base is inconsistent. A significant portion of the literature comprises expert opinions, narrative reviews, consensus recommendations, and extrapolations from obesity and MBS care, rather than randomised trials specifically examining dietetic pathways in GLP-1 RA users [29,31,36,37,63]. Consequently, a pragmatic yet cautious conclusion is warranted. While structured dietetic care is biologically plausible, clinically coherent, and well-aligned with existing obesity-care principles, further investigation is needed to determine its optimal intensity, timing, and cost-effectiveness.

## 4. Discussion

### 4.1. Why Structured Dietetic Care Is Needed

#### 4.1.1. Nutritional Deficiency and Gastrointestinal Tolerability

Obesity itself may be associated with baseline nutritional vulnerability, as excess energy intake can coexist with poor dietary quality and micronutrient deficiencies, including iron, zinc, magnesium, calcium, vitamin D, vitamin B12 and folate [58]. Against this background, pharmacological weight loss treatment may induce predictable nutritional and behavioural challenges that generic lifestyle advice does not fully address. Factors such as reduced appetite, smaller portion sizes, nausea, food aversion and gastrointestinal symptoms may have important nutritional consequences. It can be hypothesised from this indirect evidence that without structured guidance, patients may lose weight while failing to meet protein, fibre, micronutrient and hydration requirements [10,29,33,34,37,63]. As a consequence, this may increase the risk of nutritional inadequacy and aggravate pre-existing disordered eating behaviours in vulnerable individuals. In pregnancy, there are potential nutritional implications for both maternal and foetal health.

Mild nausea, constipation, diarrhoea, reflux or early satiety are common during initiation or dose escalation and often improve over time. However, symptoms become clinically significant when they persist, limit dose escalation, reduce oral intake, compromise hydration or contribute to treatment discontinuation. Semaglutide safety data indicate that gastrointestinal complaints are the main adverse-event-related cause of discontinuation in STEP 1 trials, while expert reviews note that nausea, vomiting, diarrhoea and early satiety during titration can substantially reduce oral fluid intake and increase dehydration risk [37,46,48].

Dietetic strategies may help improve tolerance by encouraging smaller and more structured meals, slower eating, stopping at fullness, avoiding high-fat or symptom-triggering foods, maintaining hydration, gradual fibre titration and food/symptom diaries [30,32,37,46]. Persistent vomiting, dehydration, severe abdominal pain, inability to maintain intake or symptoms that remain despite dietary adjustment should prompt medical review and consideration of dose modification, delayed titration, or temporary treatment interruption [30,32,37,46].

#### 4.1.2. Preservation of Lean Mass and Physical Function

Preserving lean mass and physical function rather than mere weight loss is an important consideration during pharmacological weight loss. Obesity treatment increasingly results in weight reduction levels that may significantly alter body composition [5,9,48]. Although some loss of fat-free mass is anticipated with weight reduction, patients with sarcopenia, advanced age, frailty, or low baseline activity may require more proactive nutritional and physical activity strategies [40,41,42]. Indirect evidence from bariatric surgery also supports the importance of structured physical activity alongside nutritional care during weight-loss interventions. A systematic review and meta-analysis found that postoperative exercise training was associated with greater weight loss, fat loss, improved cardiorespiratory fitness and increased muscle strength, although it did not significantly preserve lean body mass [69]. This suggests that exercise may improve functional outcomes even when measurable preservation of lean mass is not clearly demonstrated. Furthermore, higher protein intake has been shown to mitigate the loss of lean tissue mass [43].

Evidence defining optimal protein targets specifically during GLP-1 RA therapy remains limited, and current recommendations are therefore largely pragmatic. Medication-induced appetite suppression, early satiety and gastrointestinal adverse effects may reduce both meal size and frequency, resulting in protein intake below recommended thresholds, particularly among patients consuming only one or two small meals each day. Joint advisory recommends approximately 1.2–1.6 g/kg/day of protein, while the 2026 European Association for the Study of Obesity expert consensus suggests considering a target of 1.0–1.5 g/kg adjusted body weight/day during weight loss with incretin-based therapies [31,70]. Targets toward the upper end of this range, approximately 1.2–1.5 g/kg/day, may be particularly relevant to older adults, individuals with multiple comorbidities, low baseline muscle mass or sarcopenic obesity, and those experiencing rapid weight loss [38,42]. Protein requirements should therefore be individualised according to age, renal function, body composition, comorbidities, dietary tolerance, physical activity and treatment phase.

Practical management should focus on protein-forward meal structuring, with high-biological-value protein distributed across several small meals or snacks rather than concentrated in a single meal [71]. Patients may be encouraged to consume a small portion of protein at the beginning of each meal and select nutrient-dense, readily tolerated sources such as eggs, dairy products, fish, poultry, tofu and soy [30,72]. Oral protein supplementation may be considered when requirements cannot be achieved through food alone, particularly in patients with vegetarian diets, marked appetite suppression or gastrointestinal intolerance [38]. Adequate protein intake should also be combined with resistance-based physical activity where clinically appropriate, as increasing protein intake alone may not fully prevent lean mass loss during severe energy restriction [42]. These considerations support a role for dietitians in translating broad protein recommendations into individualised, feasible and clinically appropriate dietary strategies during incretin-based treatment. Dietetic professionals are well positioned to translate these principles into practical advice on protein targets, protein distribution, meal structure, eating behaviours, resistance exercise support, and symptom-adapted dietary plans.

#### 4.1.3. Long-Term Weight Maintenance and Support During Treatment Withdrawal

Long-term weight maintenance represents a further rationale for structured dietetic and behavioural support. There is consistent direct evidence that weight regain occurs following the cessation of GLP-1 RA treatment [15,16,17,18,19]. Evidence also suggests people discontinue treatment due to adverse effects as opposed to target weight being met. Studies have shown that the human factors that increase the likelihood an individual will discontinue treatment are age >65, people of lower incomes, reduced weight loss whilst on treatment, and moderate or severe gastrointestinal symptoms [14,73]. By discontinuing treatment, people not only gain weight, but also lose the cardiometabolic benefits associated with weight loss. The dietitian’s role in this is to identify the adverse effects before a patient has discontinued therapy so they can implement strategies to mitigate them.

Valuable lessons can be learnt from the National Weight Control Registry (NWCR) [74]. In this 10-year observational study, more than 87% of participants were managing to maintain at least 10% weight loss at 5 and 10 years. Being more active, monitoring weight and keeping to a lower fat diet were associated with more positive outcomes. Similarly, following bariatric surgery, those who started weigh themselves, monitored their food intake, and made positive changes in eating patterns had a better weight loss three years after surgery. Overall, the same dietary advice whilst on treatment should be maintained when off treatment [10,15,19,36]. Guidelines suggest having well rehearsed action plans, social support, regular follow ups for at least one year, and to make small manageable changes to daily routine [6].

Another strategy to prevent weight gain associated with the discontinuation of GLP-1 RA is to follow a tapering regime. This could avoid the ‘rebound effect’ of appetite suppression. The dietitian could play an essential role in this phase, by personalising each individual’s tapering regime based on their weight trend, reported levels of hunger, and adherence to lifestyle change and any metabolic deterioration identified [73].

For long-term weight maintenance, physical activity is also essential to reaching weight loss potential [17]. Lundgren et al. reported that combining structured exercise with liraglutide resulted in greater reductions in body weight and body-fat percentage than either intervention alone, alongside improvements in HbA1c, insulin sensitivity, and cardiorespiratory fitness [75].

Structured dietary and behavioural counselling may thus complement pharmacotherapy by equipping patients with transferable skills, including meal planning, prioritising protein intake, managing hunger and satiety cues, relapse prevention strategies, and sustainable approaches to physical activity. These skills may remain beneficial beyond the active treatment period, and support weight maintenance when pharmacological appetite suppression is reduced or withdrawn [16].

Furthermore, the NHS Healthier You programme for preventing diabetes has shown a 3-step, 9-month programme that incorporates exercise, stress management, and healthy eating to be very effective at preventing the onset of type 2 diabetes [76]. Initial research demonstrated a 58% reduction in the development of diabetes from lifestyle intervention compared to a 31% reduction when using metformin. This programme runs across 9 months and involves both individualised and group classes. The group classes provide an element of social support, which NICE also recognise as being key in obesity treatment success [6]. A similar programme could be used during GLP-1 RA treatment, in the tapering phase and when discontinued to maximise the longevity of its effects on obesity.

#### 4.1.4. Pregnancy and GLP-1 Agonist

Pregnancy and preconception planning require specific consideration in patients receiving GLP-1 receptor agonists or dual incretin therapy. Preclinical animal studies have identified adverse foetal effects, including growth restriction and skeletal abnormalities [77,78]. Although human data remain limited, current manufacturer guidance recommends discontinuing treatment before conception. Semaglutide should be stopped at least two months before a planned pregnancy, whereas Tirzepatide should be discontinued at least one month beforehand because of their prolonged half-lives [6,79]. Treatment should also be stopped if pregnancy occurs, followed by prompt medical review and transition to appropriate antenatal care.

Dietetic support may be particularly important during this period, as appetite suppression, reduced dietary intake and gastrointestinal adverse effects may compromise nutritional adequacy at a time of increased maternal and foetal nutritional requirements. Assessment should include dietary quality, hydration status, weight trajectory and the risk of micronutrient inadequacy, particularly involving folate, iron, calcium and vitamin D. Patients planning pregnancy, or those who become pregnant while receiving GLP-1 receptor agonist therapy should therefore receive timely medical and dietetic review to support adequate nutrition, appropriate supplementation and healthy foetal growth.

### 4.2. Lessons from MBS

MBS serves as an effective comparator because it integrates nutritional care into the obesity treatment pathway. The primary insight is not that users of GLP-1 RA should entirely adopt MBS protocols. The risk mechanisms differ; MBS may lead to reduced intake, anatomical restriction, altered absorption, and procedure-specific deficiencies, whereas GLP-1 RA therapy primarily reduces intake and may cause gastrointestinal symptoms without surgically induced micronutrient malabsorption [10,20,21,22,23,29,37,61].

Nonetheless, several principles from MBS are applicable and transferable. Firstly, nutritional assessment should be conducted prior to treatment, rather than only after issues arise. Secondly, patients should be provided with structured education regarding protein intake, hydration, dietary quality, micronutrient deficiencies, and symptom management. Protein optimisation and resistance-based physical activity advice are relevant to both surgical and pharmacotherapy pathways to minimising loss of lean mass and functional decline, but targets should be individualised. Lastly, long-term follow-up and behavioural support is essential, as weight management continues beyond achieving maximal weight loss [20,21,22,23,24,25,26].

On the other hand, some components of MBS dietetic pathways are not directly transferable to GLP-1 RA care. Routine lifelong multivitamin and mineral supplementation, procedure-specific biochemical monitoring and lifelong surveillance are central to many bariatric pathways because surgery can reduce intake, alter gastrointestinal anatomy and, in some procedures, impair nutrient absorption. Monitoring may therefore be tailored according to the type of operation, including assessment of nutrients [27,28,29,30,31,32,33]. These principles should not be applied automatically to all patients receiving GLP-1 RA therapy, as pharmacological treatment does not bypass the stomach or small bowel and does not cause surgically induced malabsorption. Instead, micronutrient assessment and supplementation should be targeted according to nutritional risk, dietary intake, symptoms, comorbidities, previous bariatric surgery and confirmed biochemical deficiency.

Similarly, staged postoperative dietary progression and management of surgery-specific complications are largely unique to MBS. Patients undergoing bariatric surgery may require a structured transition from liquids to puréed, soft and solid foods, as well as dietetic support for complications such as dumping syndrome, anastomotic problems, strictures, procedure-related vomiting, severe malabsorption and post-surgical food intolerance [21,22,23,24,25,26]. These issues are not generally applicable to GLP-1 RA therapy. Dietetic support and nutritional care in GLP-1 RA pathways should instead focus on medication-related effects such as appetite suppression, early satiety, gastrointestinal symptoms and food aversion [10,11,12,34,36,37,44,45,46,47,56,58]. Therefore, the main transferable lesson from MBS is not the direct adoption of bariatric protocols, but the principle that substantial weight-loss interventions require structured nutritional assessment and risk assessment, individualised dietary advice, symptom management, targeted monitoring and long-term support.

Another important lesson from bariatric surgery is that reduced intake does not necessarily translate into improved dietary quality. Studies following MBS suggest that patients may continue to consume broadly similar types of foods after surgery, with limited change in overall eating habits or food preferences [80,81]. This challenges the assumption that weight-loss interventions automatically produce healthier dietary patterns. For patients receiving GLP-1 RA therapy, a similar issue may arise; appetite suppression may reduce portion size and total energy intake without necessarily improving the nutritional quality of the diet. This reinforces the need for dietetic input to support nutrient-dense food choices, adequate protein and fibre intake, and sustainable long-term eating behaviours.

MBS pathways also underscore the significance of multidisciplinary care. Dietitians, surgeons, physicians, nurses, psychologists, physiotherapists, and primary care clinicians all play roles in ensuring postoperative safety and long-term outcomes. A similar model, tailored to pharmacotherapy, could involve prescribers initiating treatment and monitoring medication response, while dietitians focus on screening, nutritional adequacy, body composition risk, gastrointestinal symptom strategies, eating behaviour, and maintenance planning [31,61,64,65,66,67,68].

### 4.3. Proposed Role of the Dietitian Before, During and After Treatment

The dietitian’s role extends beyond offering generic lifestyle advice to include translating appetite suppression into nutritionally adequate eating patterns, supporting protein intake and lean mass preservation, managing gastrointestinal symptoms, and preparing patients for long-term weight maintenance during dose reduction or discontinuation [61].

Initial screening before treatment should include full dietetic assessment to ascertain nutritional risk by looking at dietary quality, protein intake, hydration, gastrointestinal symptoms, previous MBS, micronutrient risk, disordered eating behaviours and sarcopenia risk. Validated, quick screening tools such as SCOFF and SARC-F could be used to identify those at risk of eating disorder and sarcopenia [35,54,57]. During treatment, dietetic input should focus on maintaining nutritional adequacy, managing gastrointestinal symptoms, supporting protein and fibre intake, and preserving lean mass through an individualised dietary plan. In addition, during treatment, dietitians act as a support and can reinforce positive habits such as eating small, regular meals focusing on patient preference, whilst discouraging harmful eating patterns such as emotional eating and long periods of fasting [31]. After treatment interruption or discontinuation, dietetic care should focus on weight maintenance, relapse prevention and early identification of weight regain. Closer monitoring during treatment initiation may help patients manage adverse effects proactively and remain engaged with therapy.

**Figure 2:**
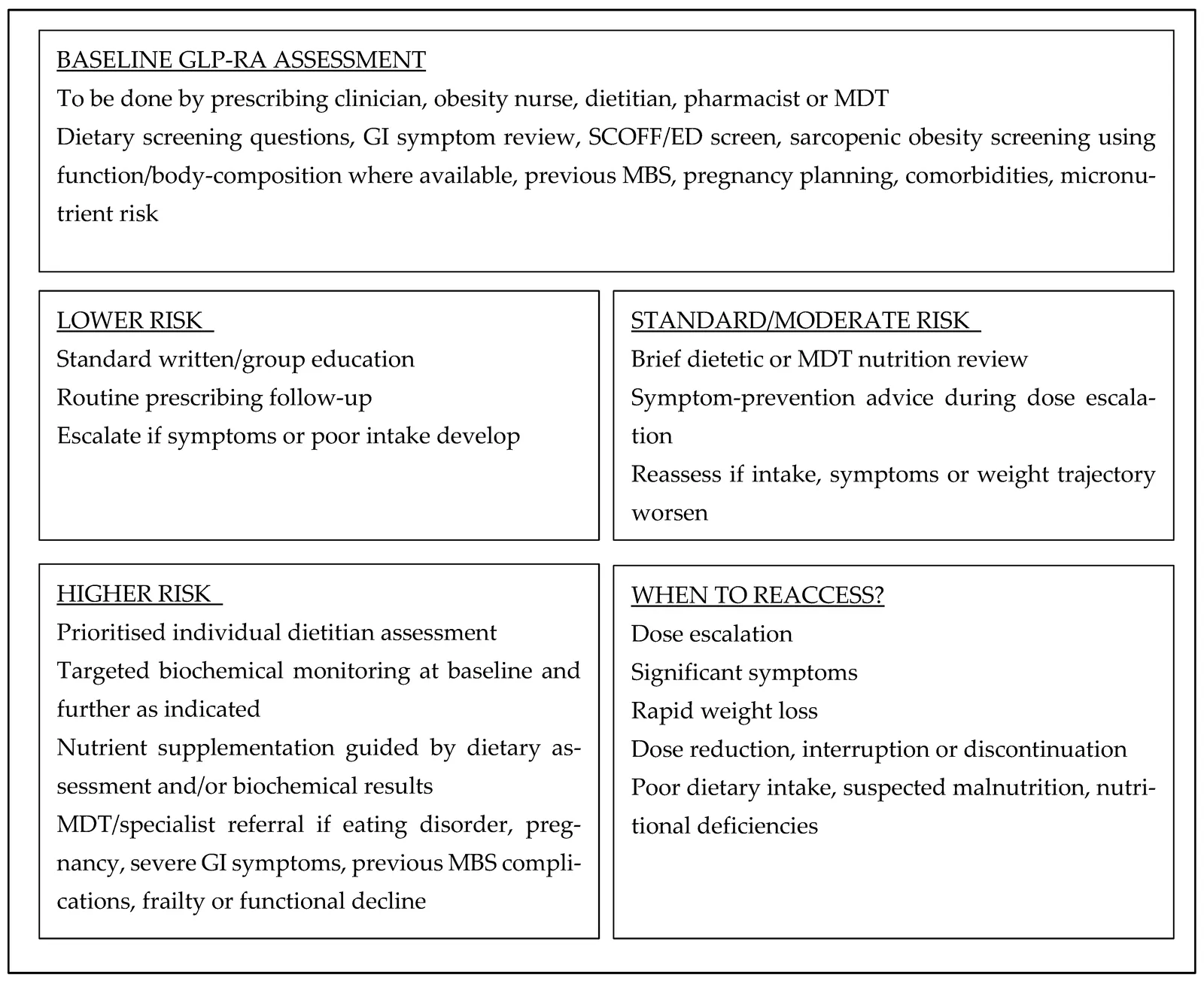
Proposed risk-stratification framework for dietetic care during GLP-1 RA therapy.

As comparative evidence has not established the most appropriate monitoring schedule for GLP-1 RA obesity pharmacotherapy, follow-up should be tailored to the stage of dose titration, severity of symptoms, nutritional risk, comorbidities, and access to care [73]. We propose reviewing patients at each dose escalation, usually every four to six weeks, and whenever they experience significant symptoms, rapid weight loss, or a dose reduction, temporary interruption, or discontinuation of treatment. Each review should include a structured risk assessment to classify the patient into one of three risk categories and guide the intensity of ongoing support. After a stable maintenance dose has been reached, routine reviews may generally be spaced every three to six months, with additional contact arranged when required to address symptoms or unexpected changes in weight. A proposed risk-stratification algorithm is shown in Figure 2, Table 1 and Table 2, while the practical dietetic care pathway across these phases is summarised in Table 3. This framework is informed by evidence on dietary intake, gastrointestinal symptoms, lean mass preservation, weight regain, eating-disorder risk, disorder risk and established MBS dietetic care pathway, and should be adapted according to local service capacity, affordability and healthcare equity [10,20,21,22,23,24,25,30,31,32,33,34,37,41,42,51,61,64,65,66,68,70].

### 4.4. Implementation Challenges

Several challenges in implementation warrant consideration. The capacity for dietetic services is known to be limited in numerous health systems globally, owing to factors such as service commissioning and resource allocation. Formal health-economic evaluations specifically assessing dietitian integration within GLP-1 RA obesity treatment pathways remain limited, and a full cost-effectiveness analysis was beyond the scope of this narrative review. Nevertheless, resource implications should be considered when designing services, particularly in healthcare systems with limited dietetic and multidisciplinary capacity. In related UK service models, broader multidisciplinary tier 3 obesity-management services in primary care settings have been reported to cost approximately £900–£1250 per patient per year for the full therapist team, although these data do not isolate the cost of dietetic input alone [82]. In the DiRECT/Counterweight-Plus intervention reported by Xin et al., practitioner input was delivered by a practice nurse or dietitian, with participants receiving approximately 23 practitioner visits over 2 years, each lasting 25–35 min; practice nurse/dietitian visits cost approximately £506 per participant over 2 years in 2018 UK prices [83]. These indirect data suggest that staff time, number of contacts and intervention intensity are important drivers of cost. Consequently, a pathway necessitating a comprehensive dietitian assessment for each patient may be impractical. To optimise the use of dietetic expertise, strategies such as risk stratification, group education, digital resources, and escalation criteria may be required [31,52,65]. Therefore, our proposed pathway of risk stratification may guide resource-limited situations. Future studies directly evaluating the cost-effectiveness of dietetic input within GLP-1 RA care pathways are warranted to better guide risk-stratified service design.

Another challenge is the absence of standardised monitoring thresholds. While MBS guidelines offer procedure-specific biochemical monitoring schedules, equivalent pathways for GLP-1 RA monitoring have not yet been established [21,22,23,24,25]. Directly applying MBS protocols to pharmacotherapy could result in unnecessary testing for low-risk patients, whereas inadequate monitoring of high-risk patients might lead to undetected, preventable nutritional issues. Therefore, future pathways should differentiate between universal education and targeted monitoring.

Lastly, the commercial and private prescribing environments may lead to fragmented care. Some patients may obtain medication without integrated dietetic support, while others might receive lifestyle advice that is too generic to effectively address gastrointestinal symptoms, protein adequacy, body composition, or the risk of eating disorders. As the use of these medications expands, establishing clear referral criteria and shared-care models among prescribers, dietitians, primary care providers, and specialist obesity services will be crucial.

## 5. Limitations and Future Research

This narrative review possesses several limitations. Firstly, despite employing a structured search strategy, the review was not conducted as a systematic review and therefore did not incorporate a formal risk-of-bias assessment, independent duplicate screening, or meta-analysis. As a result, selection bias cannot be ruled out entirely. Secondly, some evidence concerning the dietitian’s role is sourced from trial protocols, professional commentary, or country-specific healthcare models, which may restrict direct generalisability across various obesity services and health systems. Furthermore, there are no randomised controlled trials that directly evaluate the effect of structured dietetic care on clinical outcomes in patients receiving GLP-1 RAs. Moreover, the proposed risk-stratification framework is an evidence-informed model developed by the authors to translate the available literature into a practical clinical approach. It has not been prospectively validated and piloted in clinical practice. These limitations underscore the need for prospective studies evaluating structured dietetic care within pharmacological obesity treatment pathways.

Future research should extend beyond demonstrating weight-loss efficacy to evaluate the nutritional care model associated with pharmacotherapy. Key priorities include prospective studies on dietary intake, protein adequacy, micronutrient status, hydration, gastrointestinal symptoms, body composition, and muscle function during GLP-1 RA and dual incretin therapy. Trials should investigate whether structured dietetic care enhances adherence, treatment tolerance, nutritional status, lean mass preservation, and long-term maintenance.

Additionally, research is required to determine which patients derive the most benefit from dietetic input. Specific attention should be given to older adults, patients with sarcopenic obesity, those with previous MBS, individuals with a history of eating disorders, and patients experiencing significant gastrointestinal symptoms, rather than treating them as incidental subgroups [27,29,30,50,51,52]. Standardised risk tools could facilitate the identification of patients who require enhanced dietetic reviews at baseline.

## 6. Conclusions

GLP-1 RAs have significantly advanced obesity management. However, emerging evidence suggests that pharmacotherapy alone is unlikely to achieve sustainable long-term outcomes. Studies consistently highlight nutritional and behavioural challenges associated with these therapies, including reduced dietary intake, gastrointestinal adverse effects, loss of lean body mass, and weight regain following treatment discontinuation. Dietitians are well placed to address these challenges through nutritional assessment, individualised dietary counselling, symptom management, protein optimisation, behavioural support, and strategies to promote long-term weight maintenance, such as resistance exercise training and learnt relapse prevention strategies. Despite the growing recognition of their potential role, there remains an absence of direct evidence to guide the integration of dietitians into GLP-1 RA treatment pathways, and no standardised framework currently exists to support clinical practice.

The risk-stratified matrix proposed in this review offers a pragmatic model for delivering personalised dietetic care while making efficient use of healthcare resources. Healthcare systems should consider funding integrated dietitian-led obesity services alongside pharmacological treatment. Prescribers should incorporate baseline nutritional assessment into routine care and refer patients for early dietetic review, particularly for patients with previous bariatric surgery, poor intake, significant gastrointestinal symptoms, frailty, sarcopenia risk, micronutrient deficiency risk or planned treatment discontinuation. Finally, future research should evaluate dietitian-led GLP-1 RA care pathways in randomised controlled trials comparing intensive dietetic support with standard care for outcomes such as nutritional adequacy, lean mass preservation and long-term weight maintenance. This will provide a stronger evidence base by which to produce new professional guidelines in treatment.

## Figures and Tables

**Table 1:** Proposed risk-stratification framework for dietetic care during GLP-1 RA therapy.

Risk Group	Patient Characteristics and Clinical Risk Factors	Nutritional and Dietetic Risk Factors	GLP-1 RA Therapy-Related Factors	Escalation Triggers
Lower risk	No history of previous MBS; no eating disorder history; no major comorbidity affecting dietary intake or nutrient absorption; younger or functionally independent adult.	Good baseline dietary quality. No micronutrient deficiency.	No significant GI symptoms during dose escalation. Maintaining good dietary quality and eating patterns on reduced intake.	Escalate if persistent nausea or vomiting, poor intake, rapid weight loss, constipation or diarrhoea affecting intake, suspected deficiency or patient concern, severe deficiency or treatment discontinuation due to tolerability.
Moderate risk	History of constipation, diarrhoea, reflux or irritable bowel syndrome; comorbidities that do not affect nutritional intake or absorption; mild frailty.	Reduced baseline dietary quality; inconsistent protein intake; restrictive dietary pattern. Check for and correct nutritional deficiencies.	Early symptoms during dose escalation.	Escalate to individual dietitian review if intake remains poor, symptoms persist, weight loss is rapid or excessive, nutritional deficiency is suspected or treatment tolerance is compromised, severe deficiency or treatment discontinuation due to tolerability.
Higher risk	Previous MBS; older adult with functional decline; suspected sarcopenic obesity; confirmed or suspected micronutrient deficiency; active or previous eating disorder; pregnancy planning or pregnancy; CKD or other comorbidity affecting nutrition.	Significant or prolonged poor baseline dietary quality; inconsistent protein intake; restrictive dietary pattern. Check for and correct nutritional deficiencies. Screen for eating disorders. Onward referral to psychologist if concerns. MDT assessment of function. Liaise with specialist healthcare professionals regarding comorbidities that affect nutrition. Discuss pregnancy planning.	Persistent gastrointestinal symptoms. Early weight regain after dose reduction or discontinuation.	Urgent review if persistent vomiting, dehydration, inability to meet nutritional requirements, rapid functional decline, suspected eating-disorder relapse, pregnancy, severe deficiency or treatment discontinuation due to tolerability.

**Table 2:** Screening and Monitoring Pathway.

Group	Suggested Screening Criteria at Baseline	Action	Initiation of GLP-1 RA Therapy	Monitoring During GLP-1 RA Therapy	Escalation Triggers
All	Weight, height, weight history		Assess dietary quality and eating patterns Assess gastrointestinal symptoms Repeat biochemical laboratory tests as appropriate	Continue to assess dietary quality and eating patterns and symptoms Monitor monthly until patient is established on GLP-1 RA and there are no concerns. Consider moving to three-monthly monitoring. For those where there are concerns regarding dietary quality, eating behaviours, rate of weight loss, continue with at least monthly monitoring.	Early symptoms during dose escalation—individual dietitian review if intake remains poor, symptoms persist. Urgent review if persistent vomiting, dehydration, inability to meet nutritional requirements, rapid functional decline, suspected eating disorder relapse, pregnancy, severe deficiency or treatment discontinuation due to tolerability.
	Assessment of baseline dietary quality and eating patterns	Advice on dietary intake and eating patterns			
	Biochemical laboratory tests—full blood count, ferritin, folate, vitamin D where appropriate	Correction of nutritional deficiencies			
	Screening for eating disorders using SCOFF	Refer those with active eating disorders for specialist psychology assessment.			
Previous MBS Health conditions that affect nutrition, e.g., coeliac disease, inflammatory bowel disease, cystic fibrosis, renal disease.	Biochemical laboratory tests—full blood count, ferritin, folate, vitamin B12, vitamin D, calcium, (and zinc, copper and selenium post MBS).	Correction of nutritional deficiencies			
Older age Frailty or sarcopenia risk.	Screen for decreased function e.g., chair to stand. Use validated screening tools such as SARC-F.	Refer those at risk for MDT assessment			

**Table 3:** Proposed dietetic care pathway for patients receiving GLP-1 receptor agonist therapy.

Treatment Phase	Dietetic Focus	Key Components of Dietetic Care	Patients Who May Require Closer Dietetic Input
Before treatment	Baseline nutritional assessment and preparation for pharmacological weight loss.	Assess dietary quality and eating pattern, protein intake, fibre intake and hydration. Review weight history, previous dieting, supplement use and gastrointestinal symptoms. Identify previous MBS, frailty, sarcopenia risk and current or previous disordered eating behaviours. Develop a personalised dietary plan to support adequate protein, fibre, fluid and micronutrient intake during appetite suppression. Baseline biochemical assessment for microdeficiency and targeted supplementation plan. Counselling on anticipated GI symptoms, dose escalation at the patient’s pace rather than fixed, psychological challenges.	Previous MBS. Restrictive dietary patterns. Symptoms suggestive of deficiency. Frailty or sarcopenia risk. Older age. Current or previous disordered eating behaviours. Patients with certain health conditions, e.g., coeliac disease, inflammatory bowel disease, cystic fibrosis, renal disease.
During treatment	Maintenance of nutritional adequacy during weight loss and dose escalation.	Provide counselling on food and nutrition, meal structure and protein prioritisation. Encourage adequate hydration. Support management of nausea, vomiting, diarrhoea, constipation, food aversion and early satiety. Adjust dietary advice during dose escalation and periods of reduced intake. Reinforce lean mass preservation through adequate protein intake and resistance exercise advice. Ongoing biochemical or dietary assessment for micro/macronutrient deficiency and targeted supplementation plan.	Significant gastrointestinal symptoms. Poor dietary intake. Difficulty meeting protein, fibre or fluid targets. Older age, frailty or sarcopenic obesity risk. Food aversion or intolerance during dose escalation. Excessive or inadequate weight loss.
After dose reduction, interruption or discontinuation	Weight maintenance and relapse prevention.	Support strategies to manage increased hunger and larger portion sizes. Reinforce structured meal planning and dietary self-monitoring. Review protein and fibre intake during weight-maintenance phases. Develop relapse-prevention strategies for return of previous eating patterns. Identify early weight regain or nutritional deterioration requiring medical review or multidisciplinary input.	Patients stopping treatment. Patients reducing dose or restarting after interruption. Weight regain after withdrawal. Hunger recurrence. Loss of dietary structure or difficulty maintaining behavioural changes.

## Data Availability

No new data were created or analysed in this study. Data sharing is not applicable to this study.

## Supplementary Materials

The following supporting information can be downloaded at: https://www.mdpi.com/article/10.3390/nu18162643/s1, Table S1: A summary table of key articles used to answer our review question.

## Abbreviations

Abbreviations used in this manuscript are as follows:

MBS	Metabolic and Bariatric Surgery
ASMBS	American Society for Metabolic and Bariatric Surgery
BOMSS	British Obesity and Metabolic Specialist Society
EAES	European Association for Endoscopic Surgery
ESPEN	European Society for Clinical Nutrition and Metabolism
NICE	National Institute for Health and Care Excellence
GLP-1	Glucagon-Like Peptide-1
GLP-1RA	Glucagon-Like Peptide-1 Receptor Agonist
GIP	Glucose-Dependent Insulinotropic Polypeptide
