# Supplementary material for: Do Patients Receiving GLP-1 Receptor Agonists for Weight Loss Require Structured Dietetic Care? Lessons from Metabolic and Bariatric Surgery (MBS) Pathways
Source: Nutrients. 2026 Aug 13;18(16):2643. doi: 10.3390/nu18162643 (PMC13516109; doi:10.3390/nu18162643)
Supplement: Supplementary file 1 [file nutrients-18-02643-s001.zip › nutrients-4461921-supplementary.pdf]

Author / Year	Study type / design	Treatment/pathway focus	Key findings
Sibal et al., 2025	Narrative review	All GLP1RA for weight loss	-GLPRA frequently caused inadequate protein intake and occasional sarcopenia<br>-most studies do not distinguish between fat mass and skeletal muscle mass separately so it is difficult to extrapolate subgroups at higher nutritional risk
Aronne et al., 2024	SURMOUNT4 RCT	Tirzepatide continuation vs withdrawal	Participants who discontinued tirzepatide regained weight, while continued therapy maintained or augmented weight loss. Supports the need for long-term maintenance strategies during dose reduction or discontinuation
Rubino et al., 2022	STEP4 RCT	Semaglutide discontinuation	Weight regain occurred after withdrawal of semaglutide with cardiometabolic benefits moving back towards baseline. Supports the need for ongoing behavioural and nutritional support to prevent the comorbidities associated with obesity.
Ryan et al., 2024	SELECT RCT	The long term weight loss effects of semaglutide	Over the 208 week study period, those randomized to semaglutide group lost -10.2% compared to -1.5% in placebo group. 12% of previously obese patients reached healthy BMI compared to 1.2% in the placebo group.
Al-Najim et al., 2025	Narrative review	Using nutrition to ensure improved patient outcomes on GLP1RA	Some individuals were consuming <800kcal/day which when prolonged can cause sarcopenia, metabolic dysfunction and poor adherence rates to treatment.
Butsch et al., 2025	Observational retrospective study	metformin vs GLPRA + metformin	Individuals who had GLPRA and metformin in combination were significantly more likely to have nutritional deficiencies. The chance of this being detected was reduced by approximately 30% if the individual was seen in the 12 months of initiation by a dietitian
Bellicha et al., 2021	Systemic review and meta-analysis	Exercise training programmes pre and post bariatric surgery	Postoperative exercise was associated with greater weight loss, fat loss, improved cardiorespiratory fitness and increased muscle strength, although not clear preservation of lean mass.
Spreckley et al., 2026	Systemic scoping review (across 5 databases)	Nutrition strategies during incretin therapy	Found limited direct evidence to guide nutritional care during GLP-1 RA or dual incretin therapy. Highlighted lack of standardised nutrition protocols, limited dietitian involvement and inadequate reporting of nutritional outcomes. Supports the evidence gap and need for further structured research
Moetaz et al., 2025	Systematic review and meta-analysis	Dietitian role in GLP-1 RA trials	Examined dietitian involvement within GLP-1 RA trials. Supports the point that structured lifestyle or dietetic support is commonly embedded but emphasises the importance of having registered dietitians included in trials so we can better understand the importance of their role in medical nutrition therapy.

Lundgren et al., 2021	Randomised control trial	Liraglutide and exercise alone vs in combination vs placebo for weight loss	Combined exercise and liraglutide produced greater improvements in body weight, body-fat percentage and cardiometabolic outcomes than either intervention alone. Supports combining pharmacotherapy with structured physical activity.
Alexa et al., 2026	Narrative review	Micronutrient deficiencies in obesity	Highlighted that obesity can coexist with micronutrient deficiencies despite excess energy intake. Supports baseline nutritional assessment before pharmacological weight-loss therapy.
Sievenpiper et al., 2025	Scoping review	Nutritional and lifestyle recommendations for those on GLP1RA	Provide recommendations for before during and discontinuation of GLP1RA. Suggests dose adjustment for gastrointestinal adverse effects, gives nutritional guidance and discontinuation safeguards.
Wharton et al., 2025	Phase 3b randomized control trial	Dose dependent weight change in once weekly semaglutide treatment	7.2mg of semaglutide weekly participants had greater weight reductions and reduction in waist circumference than 2.4mg and placebo. Gastrointestinal adverse events were more common with semaglutide 7.2mg however, in large these were not serious (6.8%).

Table S1: A summary table of key articles used to answer our review question
